# Application of artificial intelligence lesion labeling system-assisted endoscopic submucosal dissection for the treatment of esophageal lesions in a low-volume center: a prospective cohort study

**DOI:** 10.1097/JS9.0000000000002748

**Published:** 2025-06-20

**Authors:** Bing Li, Yue-Lun Dong, Jing-Yi Liu, Wei-Min Tan, Dong-Li He, Zhi-Peng Qi, Hon-Ho Yu, Qiang Shi, Zhong Ren, Ming-Yan Cai, Shi-Lun Cai, Bo Yan, Yun-Shi Zhong

**Affiliations:** a Endoscopy Center, Zhongshan Hospital of Fudan University, Shanghai, China; b School of Computer Science, Shanghai Key Laboratory of Intelligent Information Processing, Fudan University, Shanghai, China; c Endoscopy Center, Xuhui Hospital, Zhongshan Hospital of Fudan University, Shanghai, China; d Department of Gastroenterology, Kiang Wu Hospital, Macau SAR, China

**Keywords:** artificial intelligence, early esophageal cancer, endoscopic submucosal dissection, lesion delineating

## Abstract

**Background and aims:**

Multiple artificial intelligence (AI) systems have been developed to assist with endoscopic diagnosis. We established the first real-time AI lesion-labeling system to assist in delineating lesion margin during endoscopic submucosal dissection (ESD). We aimed to further validate the efficacy of this system in improving histological complete resection rate especially for beginners in low-volume centers.

**Methods:**

We performed this prospective cohort study in two endoscopy centers in Shanghai, China from January 2021 to December 2022. Eligible patients in a low-volume center equipped with real-time AI lesion labeling system were recruited consecutively to the AI group and underwent ESD performed mainly by beginners, while participants in a high-volume center underwent conventional ESD performed by experienced endoscopists. The primary outcome was complete lateral resection rate according to pathological examination of the specimen resected. Secondary outcomes were *en*-bloc resection rate, procedure duration, specimen diameter and complication rate.

**Results:**

174 patients (200 lesions) were recruited into the AI-assisted ESD group. With the use of lesion-margin labeling system, 90.0% (180/200) of ESD cases achieved negative lateral margins. The en bloc resection rate was 98.5% (197/200), and the histological complete resection rate was 87.5% (175/200). 181 patients (202 lesions) received conventional ESD in a high-volume center. There was no significant difference between AI-assisted and conventional ESD group regarding the rate of complete lateral resection (90.0% [180/200] vs 92.1% [186/202], *P* = 0.465). Total procedure duration (min) was significantly longer in AI-assisted group (82 [54,106]) compared with conventional group (49 [30,63], *P* < 0.001).

**Conclusion:**

AI lesion-labeling system showed reliable safety and efficacy in assisting to identify the margin of lesions before ESD, and potential to improve complete lateral resection rate of ESD for esophageal lesion in low-volume endoscopy centers.

## Introduction

Esophageal cancer is the sixth leading cause of cancer-related death worldwide, and esophageal squamous cell carcinoma (ESCC) is the most common histological type in East Asian countries^[1]^. Endoscopic submucosal dissection (ESD) has been recommended as the optimal strategy for most esophageal squamous cell superficial lesions with minimal risk of lymph node metastasis, mainly to achieve curative resection less invasively and more economically, with accurate pathologic staging. Endoscopic resection requires accurate delineation of the tumor extent to achieve complete excision, since positive margin of surgical specimens has been proven to be a significant risk factor of recurrence^[2]^. However, the visual identification of dysplasia and early esophageal cancer is highly subjective and requires a lot of technical learning and accumulated experience. Low volume of the center and lack of available expertise were significantly linked with lower R0 and en bloc resection rates^[3,4]^. A 68.5–81.8% rate of R0 resection in esophageal ESD was reported in low volume centers, which was lower than the recommended goal and largely attributed to inaccurate identification of lesion boundaries^[4]^. Thus, it is of great importance to introduce auxiliary systems to compensate for suboptimal resection irrespective of the competence of the endoscopists.

Multiple AI systems using deep learning and convolutional neural network with visual or audio notices for endoscopists have been developed and evaluated in clinical practice^[5,7]^. The primary tasks required from these systems were real-time detection and characterization of the lesion, which provided the endoscopists with localization, differential diagnosis and depth-prediction information^[8]^. Based on the existing systems focusing on the process of screening and quality control, the integration of delineation algorithms has showed the potential to demarcate the lesion margin precisely, thus simplifying the subsequent resection procedure. Application of AI in delineating the margin of early ESCC and precancerous lesions has rarely been investigated and evaluated in complicated real clinical settings, but is undisputed to be a remarkable step towards implementation of AI for improving endoscopic treatment outcome.

We designed this pilot study to evaluate the safety and efficacy of AI lesion labeling system in real clinical settings of ESD. We compared complete lateral resection rate of ESD conducted in a low-volume center equipped with this AI system and a high-volume center with conventional equipment, to evaluate its auxiliary value in lesion delineation. Based on the results, we aimed to provide high-quality evidence for wider application of this cutting-edge operation assisting system, especially for beginners from low-volume centers.

## Methods

### Study design

This was a prospective cohort study conducted in two endoscopy centers in Shanghai, China. A Hospital was a low-volume center (<150 esophageal ESD performed per year) with a large percent of nonexpert ESD operators. Therefore, we equipped the established AI lesion labeling system at this center to evaluate its efficacy in assistance to improve procedure outcome especially for ESD beginners. B Hospital was an internationally high-volume center (>400 esophageal ESD performed per year) staffed with already experienced endoscopists, thus enrolled as control group, where conventional ESD was performed.

The study was conducted in accordance with the Principles of Good Clinical Practice and the Declaration of Helsinki. The study protocol was approved by the Ethics Committee of both A and B Hospital, and was registered with Chinese Clinical Trial Registry(https://www.chictr.org.cn/, ChiCTR2000040072). The work has been reported in line with the STROCSS criteria^[9]^. All authors had access to the study data and reviewed and approved the final manuscript.

### Study population

Inclusion criteria were age ≥18 years, endoscopic or biopsy diagnosis of esophageal squamous cell carcinoma or precancerous lesion, with a depth prediction of intramucosal cancer (cT1a) or submucosal slightly invasive cancer (cT1b-SM1), and no lymph node or distant metastasis as indicated by imaging examination, which were eligible for ESD indications as recommended by Japanese Society of Gastroenterology guidelines^[2]^. The exclusion criteria were as follows: (i) recurrent esophageal lesion; (ii) with a history of esophagectomy or organic esophageal diseases; (iii) high American Society of Anesthesiologists classification greater than III; (iv) pregnant or lactating women; (v) inability to understand or unwillingness to sign a written informed consent; (vi) any other situations inappropriate for treatment as judged by investigators.

**HIGHLIGHTS**

- We developed a real-time artificial intelligence system to delineate the margin of early esophageal lesions and assist in ESD.
- A prospective observational study validated the safety and efficacy of the system in clinical practice.
- The complete lateral resection rate of ESD in AI-equipped low-volume center was non-inferior to that of conventional ESD performed in high volume-center, significantly higher than general low-volume centers as reported in the past.

### Model architecture

Our AI system for (Narrow Band Imaging(NBI) esophageal segmentation has been developed over the years, and its detailed network architecture has been reported in previously published literature^[6,10-12]^. In order to broaden the scope of clinical application of our AI model, the sensitivity of this model has been enhanced. Based on the lesion segmentation maps output by the AI system, clinicians can strategically identify the surrounding mucosa at the boundary of the lesion and prevent missed detection. The optimized margins recognition model was utilized in the AI-lesion labeling system in this study. Notably, it is the system’s robust performance that enabled us to conduct a prospective cohort study.

Here, we provide a more detailed introduction to the system. This AI system was an end-to-end trainable multi-task neural network, which allows esophagus lesions to be detected and classified simultaneously by only feeding an esophagoscope image. The former part of our DNN-CAD system is the VGG-16 (conv1-conv5) model pre-trained on the ImageNet dataset. Note that using the model parameters trained on the ImageNet dataset as the initial parameters of the model is a common practice in the field of computer vision. This practice can make the model easier to converge during further training, and the performance is usually better. The parameters of VGG-16 backbone network are shared between the classification and detection tasks, which has advantages of reducing computational cost and improving the performance of screening polyps and lesions. The remaining parts of the DNN-CAD system are two independent branches for implementing classification and detection separately. The classification branch consists of a global average pooling layer and three fully connected layers.

As for the detection branch, we employ the saliency detection network to detect polyps and lesions in endoscopic images. The overall architecture of detection branch is based on VGG-16 model and includes six side output channels, which encode rich spatial information. Each side channel outputs a preliminary detection result, in which the polyps or lesions are highlighted. To integrate these preliminary detection results, we add short connections between side channels. These short connections help to fuse the high-level semantic features from deeper side channel and the low-level detail features from shallower side channel. Based on the integrated result from side channels, a convolutional layer followed by the sigmoid function is added to balance the contributions of six side outputs and produce the final detection results.

### ESD procedures and data collection

16 endoscopists with different levels of ESD experience from two study centers attended the study. Among the operators, those who had performed more than 250 ESDs were regarded high-level endoscopists, and medium level endoscopists were defined as who performed more than 50 ESDs^[13]^. Junior endoscopists were those who had performed less than 50 ESDs before the study started. The distribution of ESDs by different levels of operators involved is shown in Table 1.

**Table 1:** Clinical characteristics of patients

Variables	Conventional ESD group(n = 202)	AI-assisted group (n = 200)	*P* value
Age, median (range)	65.64(42-84)	64.95(42-86)	0.387
Sex			
Male, no. (%)	148(73.27%)	141 (70.50%)	0.537
Female, no. (%)	54(26.73%)	59(29.50%)	
ASA, no. (%)			
I	34(16.80%)	26(13.00%)	0.577
II	166(82.2%)	171(85.5%)	
III	2(1.00%)	3(1.50%)	
Tumor diameter, median (range), cm	2.2(1.2,3.0)	2.3(1.1,3.0)	0.490
Tumor location, no. (%)			
Cervical esophagus	6(3.00%)	8(4.00%)	0.823
Upper thoracic esophagus	33(16.30%)	39(19.50%)	
Middle thoracic esophagus	85(42.10%)	80(40.00%)	
Lower thoracic esophagus	78(38.61%)	73 (36.50%)	
Proportion of the esophagus circumference involved, no. (%)			
<25%	60(29.70%)	43(21.50%)	0.029
25–50%	93(46.04%)	88(44.0%)	
50–75%	26(12.87%)	47(23.50%)	
75–100%	23 (11.4%)	22(11.0%)	
Macroscopic type, no. (%)			
Elevated (0-I, 0-IIa)	40(19.80%)	37(18.50%)	0.421
Flat(0-IIb)	143 (70.80%)	136(68.00%)	
Depressed (0-IIc, 0-III)	16(7.90%)	19(9.50%)	
Mixed (0-lla + llc, 0-llc + lla, 0-I + IIa)	3(1.50%)	8(4.00%)	
Patient by operator, no. (%)			0.001
High level	173(85.6%)	34(17.0%)	
Medium level	29(14.4%)	42(21.0%)	
Junior level	0(0.0%)	124(62.0%)	

The AI lesion labeling system delineates the lesion during ESD operation in real time, and presents the lesion boundary clearly by colored region on a monitor positioned adjacent and parallel to the original endoscopy monitor. The system was equipped at A Hospital, while conventional ESD were performed by endoscopists at B Hospital. Conventional ESD for esophagus lesions was performed as described before^[14,15]^.

**Figure 1.**
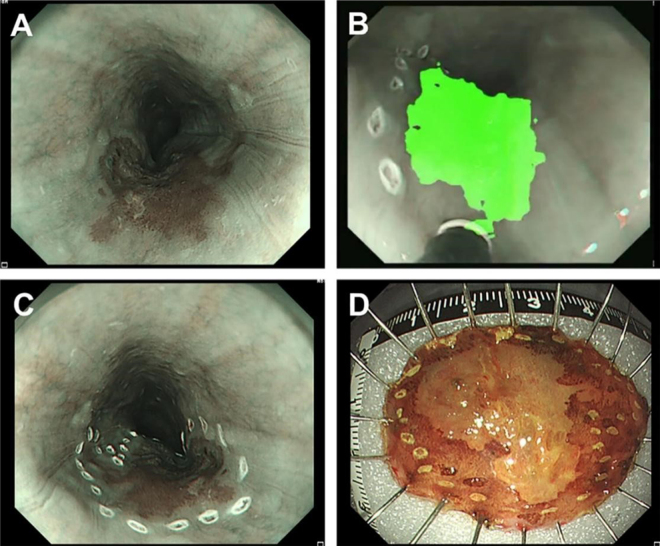
Real-time application of AI lesion-labeling system during ESD. (A) lesion located in upper thoracic esophagus was spotted. (B and C) With the help of AI system, endoscopist placed the marking dots approximately 5 mm laterally to the identified tumor edge at 2–3 mm intervals using argon plasma coagulation (APC) before incision. (D) Lugol’s solution (1.2%) was sprayed to the surface of resected specimen to make confident that the marker point completely encompasses the lesion.

Online supplementary video (available at: http://links.lww.com/JS9/E449) presents the real-time application of this AI system in assistance to marking lesion margin at the first step of ESD. This lesion was located in upper thoracic esophagus (Fig. 1A), and our AI system accurately identified and labeled the margin of the lesion. With the help of this AI system, endoscopist placed the marking dots approximately 5 mm laterally to the identified tumor edge at 2–3 mm intervals (Fig. 1B and C) using argon plasma coagulation (APC). Then, the mucosal incision line was made around the marking dots, and ESD was carried out as described in previous reports^[14,15]^. Last, Lugol’s solution (1.2%) was sprayed to the surface of resected specimen to make confident that the marker point completely encompasses the lesion (Fig. 1D). Pathological examination confirmed a complete resection free of tumor cells or dysplasia in both the basal and lateral margins.

All the resected specimens were stretched smoothly by pins on a corkboard and fixed with 10% formalin for pathological evaluation. Paraffin-embedded specimens were sectioned at 2-mm intervals. Pathologists involved were blinded to the procedure details and recorded endoscopic assessment. Two experienced pathologists from each center first evaluated the histologic outcome independently. The type of lesion and margin involvement was determined when the initial diagnosis of two pathologists were the same, otherwise the third pathologist joined for further evaluation, and together the final decision was made. All the slices from center A were brought to center B for further recheck of pathological diagnosis, and no disagreement with the final decision has been raised.

### Outcome measures

The primary outcome was complete lateral resection rate, which was defined as resection with no dysplasia or carcinoma found on any lateral margin of the specimen confirmed by histological examination by expert pathologists^[16]^.

En-bloc resection rate, R0 resection rate, procedure duration, specimen diameter and complication rate were recorded as secondary outcomes. En bloc resection was defined as resection in a single piece. R0 resection was defined as resection with tumor-free margins and no lymphatic or venous infiltration. Complete lateral resection refers to resection with tumor-free lateral margins, regardless of horizontal margins. Procedure duration was the time from initial placement of marker dots around the lesion to the time the visible vessels on cut were successfully managed in the post-ESD ulcer. Follow-up of the patients were recorded through outpatient service and telephone interview. Local residue was defined as residual neoplasia detected at the first follow-up endoscopy after ESD. Local recurrence was defined as a new lesion found by endoscopy within 1 cm of the original ESD site after 6 months. Lymph node metastases were diagnosed by pathological outcome of additional esophagectomy or imaging examination including PET and CT.

### Sample size estimation and statistical analysis

In this study testing the non-inferiority of AI-assisted ESD to conventional ESD, the optimal sample size was estimated based on the expected effect on complete lateral resection rate, which was the primary endpoint. We retrospectively reviewed the records of B Hospital, where conventional ESD procedures were performed by experienced operators, to estimate the rate of complete lateral resection of specimen for esophageal lesions of 12%. With a non-inferiority margin of 5% and 1:1 ratio between groups, a required sample size of 388 patients was then calculated considering a 1-sided error of 0.025 and β error of 0.2. To compensate for 5% predicted loss to follow-up, we aimed to include 408 patients in total.

Quantitative variables were summarized by either the mean (±standard deviation) for normally distributed data or the median and interquartile range (IQR) for skewed distribution determined by a Shapiro-Wilk test. The χ2 test was used to compare categorical variables. The t test was used for continuous and normally distributed variables, and the Mann-Whitney U test was used to compare medians if data were not normally distributed. The univariate analysis was used to adjust for confounding variables. A two-sided *P* value of 0.05 was used as the threshold for significance.

## Results

### Clinical characteristics of patients

**Figure 2.**
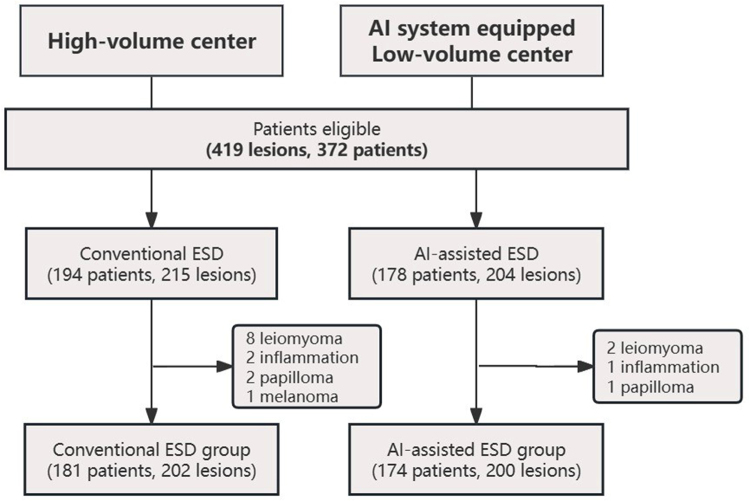
Flow diagram of the trial. A total of 419 lesions (372 patients) were enrolled originally. Among these, we excluded cases with pathological outcome other than esophageal squamous cell carcinoma or precancerous lesion (17 patients, 17 lesions). Finally, 402 lesions (355 patients) were included in study analysis. 174 patients (200 lesions) were recruited into the AI-assisted ESD group by December 2022, and 181 patients (202 lesions) into conventional ESD group by November 2021.

Between January 2021 and December 2022, a total of 419 lesions (372 patients) were enrolled in the original study. Among these, we excluded cases with pathological outcome other than esophageal squamous cell carcinoma or precancerous lesion (17 patients, 17 lesions). Finally, 402 lesions (355 patients) were included in study analysis. 174 patients (200 lesions) were recruited into the AI-assisted ESD group by December 2022, and 181 patients (202 lesions) into conventional ESD group by November 2021 (Fig. 2).

Patient demographics and lesion characteristics are presented in Table 1. The baseline characteristics including age, sex, history, tumor size and location, macroscopic type, histological outcome were well balanced between the two groups. However, proportion of the esophagus circumference involved was significantly different between AI-assisted group and conventional group.

### Outcome of AI system-assisted ESD

Table 2 summarizes the procedural outcomes of ESD for 200 early ESCCs with the use of real-time lesion-margin labeling system. The median ESD procedure time was 82 minutes (IQR 54,106 minutes). The en bloc resection rate was 98.5% (197/200), and the histological complete resection rate was 87.5% (175/200). Moreover, 90.0% (180/200) of ESD cases achieved negative lateral margins, and 195 (97.5%) of ESD cases achieved negative deep margins. Delayed bleeding after ESD developed in one patient and two minor thoracic effusion occurred. Two cases reported delayed perforation.

**Table 2:** Outcomes related to ESD for ESCC in AI-assisted group

Variables	Value (n = 200)
Procedure time, median (IQR), min	82 (54,106)
Specimen diameter, median(range), cm	3.6 (0.5–9)
*En* bloc resection, no. (%)^a^	197 (98.5%)
R0 resection, no. (%)^b^	175 (87.5%)
Negative lateral margin, no. (%)	180 (90.0%)
Negative deep margin, no (%)	195 (97.5%)
Adverse event, no. (%)	
Delayed bleeding	1 (0.5%)
Minor thoracic effusion	2 (1.0%)
Perforation	2 (1.0%)
Histological outcome, no. (%)	
Low-grade hyperplasia	25(12.5%)
High-grade hyperplasia	62(31.0%)
Squamous cell carcinoma	113(56.5%)

^a^ *En* bloc resection was defined as a specimen that was resected in a single piece.

^b^ R0 resection was defined as lateral and deep margins free from cancer and dysplasia.

In subgroup analysis stratified by operator level (Supplementary Table S1, available at: http://links.lww.com/JS9/E447), concerning complete lateral resection rate (88.20%[30/34] vs 83.30%[35/42] vs 92.70%[115/124], *P* = 0.176) and R0 resection rate(88.2%[30/34] vs 81.0%[34/42] vs 89.5%[111/124], *P* = 0.328), there was no significant difference across variate operator experience levels, given a similar constitution of pathological outcome and specimen diameter.

### Comparison of outcome between AI-assisted and conventional ESD group

The clinical outcomes are summarized in Table 3. There was no significant difference between AI-assisted and conventional ESD group regarding the rate of complete lateral resection (90.0%[180/200] vs 92.1%[186/202], *P* = 0.465). Comparison upon specimen diameter(cm)(3.6[0.5-9.0] vs 3.5[0.5-8.0], *P* = 0.682), en bloc resection rate (98.5%[197/200] vs 98.5%[199/202], *P* = 1.000), complete vertical resection rate (97.5%[195/200] vs 99.0%[200/202], *P* = 0.283), R0 resection rate(87.5%[175/200] vs 91.1%[184/202], *P* = 0.244) between two groups showed no difference as well (Table 2). However, total procedure duration(min) was significantly longer in AI-assisted group (82[54,106]) compared with conventional group (49[30,63], *P* < 0.001).

**Table 3:** Outcomes of conventional ESD and AI-assisted ESD

Variables	Conventional ESD group	AI-assisted group	*P* value
Dysplasia-free horizontal margin, no.(%)	186(92.1%)	180(90.0%)	0.465
Dysplasia-free vertical margin, no.(%)	200(99.0%)	195(97.5%)	0.283
Total procedure duration, min(IQR)	49(30,63)	82 (54,106)	0.000
Specimen diameter, cm(range)	3.5(0.5-8.0)	3.6 (0.5-9.0)	0.682
R0 resection, no.(%)	184(91.1%)	175(87.50%)	0.244
En bloc resection, no.(%)	199(98.51%)	197(98.50%)	
Adverse events, no.(%)			
Perforation	1(0.50%)	2(1.00%)	0.622
Delayed bleeding	0(0.00%)	1(0.50%)	
Pleural effusion	1(0.50%)	2(1.00%)	
Histological outcome, no.(%)			
Low-grade hyperplasia	29(14.36%)	25(12.50%)	0.247
High-grade hyperplasia	49(24.25%)	64(32.00%)	
Squamous cell carcinoma	124(61.39%)	111(55.50%)	

Concerning local residue (1.12% vs 1.06%, *P* > 0.999) and recurrence rate (0.56% vs 0.53%, *P* > 0.999), there is no significant difference between AI-assisted group and conventional ESD group. As for lymph node metastasis, no significant difference was found (1.25% vs 1.80%, *P* > 0.999). No distant metastasis or death had been observed in either group.

Moreover, considering the difference of base-line characteristics of patients between the two groups, we applied univariate analysis to adjust for proportion of the esophagus circumference involved (Table 4). There was no association between groups and the primary outcome before and after adjustment.

**Table 4:** Univariate and multivariate analyses

	Before adjustment			After adjustment		
	Odds ratio	95% confidence interval	*P* value	Odds ratio	95% confidence interval	*P* value
Conventional ESD	1(reference)			1(reference)		
AI-assisted ESD	1.292	0.649–2.572	0.466	1.217	0.595–2.490	0.591

We further conducted an analysis comparing the outcome of AI-assisted ESD by junior level performers with conventional ESD group (Supplementary Table S2, available at: http://links.lww.com/JS9/E448). There were no significant differences found in complete lateral resection rate(92.7%[115/124] vs 92.1%[186/202], *P* = 0.827) or R0 resection rate(89.5%[111/124] vs 91.1%[184/202], *P* = 0.638).

## Discussion

Squamous cell carcinoma is the most frequent histological subtype accounting for 90% of cases of esophageal cancer worldwide mainly due to its high prevalence in Eastern Asia^[1,17]^. Detection and diagnosis of early ESCC can be challenging, since it typically presents as flat and isochromatic lesions under conventional white light endoscopy^[18]^. An accurate delineation and marking of the lesion extent is essential to a complete resection as well as a rational treatment to avoid strictures formation, thus several assisting approaches have been introduced. Lugol chromoendoscopy has been conventionally used to detect ESCC, but the fading overtime and side effects caused by iodine restricted its further application during ESD^[19]^. NBI is an image-enhancing technology that improves the visualization of microvessels and mucosal color change of the esophagus, which has exhibited higher accuracy and specificity in detection and delineation of ESCC compared with Lugol chromoendoscopy^[20,21]^. The experience of the operator plays a critical role in the using of NBI to define resection margins, and AI may be uniquely poised to compensate for the lack of operator experience^[10]^. In our previous research, we have reported a computer-aided diagnosis (CAD) system to localize and identify early ESCC with high accuracy^[6]^. Here, we developed a new deep-learning based AI system to provide a real-time demarcation and visualization of margin of early ESCC under NBI endoscopy. Moreover, in this pilot study, our AI system was applied to help marking the incision margin of ESCC during ESD, showing the great potential of AI system in operation assistance more than just detection of lesions.

In the field of digestive endoscopy, several AI systems have been used in detection and delineation of early cancer^[7,22-25]^. Liu *et al*^[26]^. developed an AI model to detect and delineate margins of early ESCC under WLI endoscopy, which showed an accuracy of 84.5% in detecting lesions in external validation, and an accuracy of 95.7% for delineating margins, similar with that of expert endoscopists. However, the gold standard was defined as delineation of the lesions marked by the expert endoscopists using iodine staining and NBI as a reference instead of pathological results, which potentially overestimated the accuracy. Yuan *et al*^[27]^.have recently reported an AI system for detecting and delineating ESCC under NBI, and integrated it into the endoscopy equipment to estimate its real-time diagnostic capability, yet directly evaluation of the system was restrained by lack of rebuilding of the pathologic results. To our knowledge, no previous studies have reported on the application of AI system in more complicated real clinical settings such as endoscopic resection, nor validated the efficacy of the system directly through pathological outcome. Our real-time CAD system for esophageal neoplasm was developed on a deep learning architecture. The algorithm had been validated to have excellent per-image sensitivity, specificity and accuracy^[10,11]^. We further proposed a lesion-decoupling-based segmentation (LDS) network to precisely decouple the lesion under the contrastive learning of internal and external features of the lesion area^[12]^. Based on the lesion segmentation maps output by the AI system, clinicians can strategically identify the surrounding mucosa at the boundary of the lesion and prevent missed detection. The optimized margin recognition model was utilized in the AI-lesion labeling system in this study. Given that the algorithm used in both CAD system and lesion labeling system has been extensively verified in previous studies, including RCT clinical research, we carried out this pioneer study to demonstrate AI integration in complex real-world endoscopic resection workflows.

Our study is the first to design and apply the AI lesion labeling system in a real-time visual noticing way during ESD procedure, thus extending the function of AI to the field of complicated surgery and validate its efficacy directly through pathological examination of specimen. In AI-assisted ESD performed mainly by non-expert performers in the low-volume center, 90.0% (180/200) of ESD cases achieved negative lateral margins. The en bloc resection rate was 98.5% (197/200). Negative deep margins were achieved in 195 (97.5%) ESD cases and the histological complete resection rate was 87.5% (175/200). The median ESD procedure time was 82 minutes (IQR 54,106 minutes). Moreover, delayed bleeding after ESD developed in 1 patient and 2 minor thoracic effusion occurred, and 2 cases reported perforation, proving the safety of this system in real-world clinical settings.

In addition, endoscopists with varying experience were involved to assess on the auxiliary value of the system in compensating for lack of experience of the center and endoscopists. The study involved 2 centers and 16 attending endoscopists of different levels of ESD experience. In subgroup analysis stratified by operator level in the AI-assisted group, there is no significant difference across variate operator experience levels concerning complete lateral resection rate and R0 resection rate. On the other hand, in AI-assisted ESD group in the low-volume center, 90.0% (180/200) of ESD cases achieved negative lateral margins. The result was comparable to that described in existing studies by expert performers from advanced high volume centers (complete lateral resection rate for dysplasia or carcinoma between 87.9% and 95 %)^[16,28-31]^, and evidently outperformed those of 68.5–81.8% reported in low volume centers^[31,32]^.

An endoscopic resection with positive horizontal margins may leave the risk of local persistence/recurrence as high as 30 %, thus considered a local-risk resection^[33]^. In this case, additional treatment including endoscopic re-treatment, radiotherapy or surgery should be conducted, resulting in decrease in quality of life, difficulty of organ preservation and excess medical expenses. It is estimated that additional treatment after ESD could be as high as $33 000 in China^[34]^. Considering initial development of approximately $1389 and annual maintenance cost of $695, the investment in AI is offset by preventing even a modest number of costly recurrences, while improving equity in healthcare access.

There was no significant difference between AI-assisted and conventional ESD group regarding the rate of complete lateral resection (90.0%[180/200] vs 92.1%[186/202], *P* = 0.465). The benefit of AI labeling system in ESD is further confirmed in univariate analysis, showing that low-volume center is not an independent risk factor affecting horizontal involvement once equipped with this system. Considering contribution of medium/high-level operators in AI-assisted group, we further conducted an analysis to compare the outcome of junior level performers of AI-assisted groups with conventional ESD group. There was no significant difference found in complete lateral resection rate or R0 resection rate. The absence of medium/high-level operators in the AI group did not affect the overall results, as junior level operators in the AI group achieved outcomes comparable to Hospital B’s experts. Moreover, there was no significant difference between conventional and AI-assisted group in follow-up results, including local residue, local recurrence and lymph node metastasis. No distant metastasis or death had been observed in either group. To some extent these results could support our conclusion that AI assistance bridged the experience gap in low-volume settings.

The findings confirm that AI-assisted system significantly helps to control horizontal involvement rate within an acceptable range in the low-volume endoscopy center compared with conventional ESD performed in the high-volume center. However, this benefit may come at the potential expense of longer procedure duration in AI-assisted group in our study. This might on the one hand due to the large percent of nonexpert operators with lack of experience of ESD in AI-assisted group, as well as more time consumed to confirm and make incision along the labeling displayed on the system monitor. Given the benefit of acceptable horizontal involvement rate observed in this study, we believe that the prolongation of procedure time is a tolerable drawback.

There are certain limitations of our study. First, though the trial was carried out prospectively, it was not randomized. As an exploratory investigation, this study aimed to preliminarily evaluate the feasibility and efficacy of the newly designed AI system, especially in augmenting performance in real-world resource-constrained settings. Thus, stringent inclusion/exclusion criteria and univariate analysis was used to ensure baseline comparability. This exploratory study provides preliminary real-world evidence for AI’s value and paves the way for future large-scale randomized controlled trials. Second, we did not evaluate on the possible warning effect brought by the system itself, since the existence of this extra AI labeling system could make operators more concentrated and perform better than usual. Setting of a control group equipped with a sham system in the future would help with blind design.

In conclusion, AI lesion labeling system significantly helps to decrease horizontal involvement rate for esophageal ESDs performed in the low-volume center, and hopefully may help to promote the adoption of such complex endoscopic procedures in more low-volume centers and regions.

## Data Availability

Deidentified individual participant data and the study protocol are available upon reasonable request.

## Research registration unique identifying number (UIN)

The study was registered in Chinese Clinical Trial Registry (https://www.chictr.org.cn/, ChiCTR2000040072). The study protocol was approved by the Ethics Committee of Zhongshan Hospital Fudan University and Xuhui District Central Hospital (No. 2020-190).
